# Progression of Coronary Artery Calcification According to Changes in Risk Factors in Asymptomatic Individuals

**DOI:** 10.3390/jpm14070757

**Published:** 2024-07-16

**Authors:** Jin-Young Yoo, Se-Ri Kang, Eun-Ju Chun

**Affiliations:** 1 Department of Radiology, Eunpyeong St. Mary’s Hospital, College of Medicine, The Catholic University of Korea, Seoul 03312, Republic of Korea; 2 Department of Radiology, Wonkwang University College of Medicine and Hospital, Iksan 54538, Republic of Korea; 3 Department of Radiology, Seoul National University Bundang Hospital, Seongnam 13620, Republic of Korea

**Keywords:** coronary artery calcium, computed tomography, clinical risk factors

## Abstract

This retrospective study aimed to assess coronary artery calcium (CAC) progression in serial computed tomography measurements according to risk factor changes. In 448 asymptomatic adults who underwent CAC measurements with more than one-year intervals, CAC progression was assessed according to age, sex, variable traditional risk factors (diabetes mellitus, hypertension, hyperlipidemia, and smoking), and initial CAC score (0, 0.1–100, and >100). Univariate and multivariate logistic regression analyses were assessed for independent predictors of rapid CAC progression (ΔCAC/year > 20). During the 3.5-year follow-up, coronary artery calcifications occurred in 43 (12.8%) of 336 individuals with an initial CAC score of zero. Of 112 individuals with initial CAC presence, 60 (53.6%) had ΔCAC/year > 20. Age, male sex, body mass index, and all risk factors were significantly associated with ΔCAC/year > 20, but recently diagnosed hypertension (odds ratio [OR], 11.3) and initial CAC score (OR, 1.05) were significant independent predictors in multivariate regression analyses. CAC progression was affected by demographic and traditional risk factors; but, adjusting for these factors, recently diagnosed hypertension and initial CAC score were the most influential factors for rapid CAC progression. These findings suggest that individuals with higher initial CAC scores may benefit from more frequent follow-up scans and checks regarding risk factor changes.

## 1. Introduction

Coronary artery calcium (CAC) scoring assessed by computed tomography (CT) is a widely used and reproducible tool to guide risk stratification, as well as a powerful surrogate marker for cardiovascular disease (CVD) [1,2]. The CAC score has been extensively validated as a strong predictor of coronary events, and its superiority to risk factor-based paradigms such as the Framingham Risk Score, and individual risk factors has been demonstrated [3,4]. Therefore, the CAC score is recommended for the assessment of asymptomatic middle-aged populations, particularly intermediate-risk cohorts, by various cardiovascular society guidelines across the world [5,6].

The clinical utility of CAC scoring extends beyond initial risk assessment. Several studies have highlighted the clinical significance of CAC progression as determined by serial CT scans, with CAC progression significantly correlating with a worse prognosis compared to CAC non-progression [7,8]. Additionally, CAC progression has been linked to various traditional risk factors [3,9], some of which—such as smoking or hyperlipidemia—are modifiable; this implies that changes in these factors may impact CAC progression over time. Despite the clinical relevance of monitoring CAC progression, there remains limited evidence regarding its correlation with changes in risk factors. Consequently, current guidelines do not specify the optimal timing for follow-up CAC scanning.

Understanding the dynamics of CAC progression concerning modifiable risk factors is crucial for enhancing clinical outcomes. Early identification and management of individuals at high risk for CVD may potentially decrease the incidence of adverse cardiac events. Moreover, optimizing the utilization of CAC scoring from a public health perspective could result in more efficient allocation of healthcare resources by targeting preventive measures to those who stand to benefit the most.

Therefore, we aimed to assess CAC progression according to changes in risk factors through serial CT measurements in asymptomatic individuals.

## 2. Materials and Methods

### 2.1. Study Design and Participants

Based on the health screening data registry of the Seoul National University Bundang Hospital (SNUBH) between January 2006 and December 2012, patients who underwent serial CAC scoring at least two times with more than one-year intervals were assessed. All individuals were asked whether they had chest pain or equivalent symptoms according to the WHO Rose angina questionnaire [10]. Physicians subsequently determined asymptomatic individuals who were free of coronary artery disease based on the patients’ history, including the answers from the questionnaire. We excluded participants who had a history of myocardial infarction, stroke, percutaneous coronary intervention, or coronary artery bypass graft. Finally, we enrolled 448 self-referred asymptomatic individuals who had undergone serial CAC scoring at least two times with more than one-year intervals.

### 2.2. Risk Factor Assessment

Basic demographic data were acquired from a database maintained by the SNUBH Health Promotion Center. Medical history of myocardial infarction, angina, hypertension, stroke, diabetes mellitus (DM), family history of CVD (myocardial infarction or stroke history in first-degree relatives), current medication profile, and smoking status were systematically determined by personal interviews during the patients’ hospital visits for initial and follow-up CAC scans.

Body weight and height were also recorded during their visits, and blood samples were collected and analyzed. We recorded the mean blood pressure (BP) after the measurement of the resting BP three times with the participant in a seated position. Hypertension was defined as a systolic/diastolic BP ≥ 140/90 mmHg, respectively, or the current use of antihypertensive medication. Lipid profiles, including total cholesterol and triglycerides, and fasting plasma glucose were measured with blood sampling obtained after a 12-h fast. DM was defined as a fasting blood glucose level ≥126 mg/dL or current antidiabetic treatment. Hyperlipidemia was defined as a total cholesterol concentration of ≥240 mg/dL or current treatment with lipid-lowering drugs [11]. Current smoking was considered if the participant had smoked regularly during the previous 3 months.

### 2.3. Data Acquisition

CT data were acquired using 64-multidetector cardiac CT (Brilliance 64; Philips Medical Systems, Best, The Netherlands) with collimation of 64 × 0.625 mm and rotation time of 420 ms. CT scans used 120 kVp with variable mA according to the patient’s body weight and non-overlapping sections, with 2.5 mm slice thickness [12].

Calcium scoring was calculated using the Agatson score with a threshold of 130 Hounsfield Units (HU), with only contiguous voxels totaling ≥1 mm^2^ in the area on pre-contrast CT images. The score for an individual lesion was obtained by multiplying the lesion area with the density weighting factor determined by the maximum CT attenuation in the specified lesion. The density weighting factor was determined as follows: lesions with a peak attenuation of 130–199 HU were assigned a value of 1, those with a peak attenuation of 200–299 HU were assigned a value of 2, those with a peak attenuation of 300–399 HU were assigned a value of 3, and those with a peak attenuation value > 400 HU were assigned a value of 4 [12]. The total Agatston score was the summed score of all coronary lesions. According to the initial CAC score, patients were categorized into three groups, as follows: zero-CAC (CAC = 0), low-CAC (0.1 ≤ CAC score ≤ 100), and high-CAC (CAC score > 100) groups.

### 2.4. Statistical Analysis

Continuous parameters are presented as mean ± SD, and nominal or ordinal parameters are given as n (%). Continuous parameters among the three groups classified according to the initial CAC score were compared using one-way analysis of variance (ANOVA) with Scheffe’s post hoc test, whereas nominal data were evaluated using the chi-square or Fisher’s exact test.

Annualized progression of the CAC score (ΔCAC/year) was calculated as the last CAC score minus the initial CAC score divided by the time between the initial and last CAC scan. The group with an initial CAC score of zero was divided into two groups by examining the changes in the follow-up CAC scan: a group with continuous zero CAC scores (zero-CAC_last_) and a group with new calcium (new-CAC_last_). Those with calcium present on the initial CT scan were divided into two groups based on an annual CAC increase of more than 20 (ΔCAC/year > 20). Differences between two groups were analyzed using Student’s *t*-test for continuous data and the chi-square test for nominal or ordinal data.

Finally, univariate and multivariate logistic regression analyses were assessed for independent predictors of ΔCAC/year > 20. SPSS 17.0 package (SPSS Inc., Chicago, IL, USA) was used for all statistical analyses, and a *p*-value < 0.05 was regarded as statistically significant.

## 3. Results

### 3.1. Basic Demographics of Enrolled Asymptomatic Individuals

A total of 448 asymptomatic individuals underwent serial CAC scans two times, with an interval of more than one year. The initial CAC scores ranged from 0 to 758.3. Among them, the initial CAC score was zero in 336 patients (75.0%). The low-CAC group comprised 70 individuals (15.6%) with a CAC score of 31.6 ± 26.7, whereas 42 individuals (9.4%) with a mean CAC score of 250.6 ± 190.3 belonged to the high-CAC group. Table 1 summarizes the baseline demographic characteristics and risk factors of the study population in the three groups, classified according to the initial CAC score. Hypertension prevalence significantly increased according to the initial CAC score. Other demographic parameters—including age, prevalence of male sex, the presence of DM and hyperlipidemia, as well as statin and aspirin medication use—were significantly higher in the low- and high-CAC groups than in the zero-CAC group (all *p* < 0.05). However, these parameters were not significantly different between the low-CAC and high-CAC groups. Overall, the number of risk factors was higher in groups with CAC presence (low-CAC and high-CAC group) than in that with CAC absence (zero-CAC group).

### 3.2. CAC Progression and Changes in Risk Factors during Follow-Up

During the mean 3.5 ± 1.3 years of follow-up (range 1.0–7.5 years), the mean annual progression in CAC score was 10.0 ± 29.6. The ΔCAC/year was >10 in 87 patients (19.4%), whereas ΔCAC/year was >20 in 61 patients (13.6%). The degree of annual CAC progression was significantly higher in the group with a higher initial CAC score (*p* < 0.001).

During the follow-up period, hypertension, DM, and hyperlipidemia were newly diagnosed in 14 individuals (3.1%), 7 individuals (1.6%), and 31 individuals (6.9%), respectively. In the smoking status survey, 22 individuals (4.9%) quit smoking, whereas 6 individuals (1.3%) started smoking. These new risk factors were more frequently recorded in the group with CAC presence than in that with CAC absence in the initial CAC scan. Table 2 presents CAC progression and changes in risk factors during the follow-up period among the three groups, classified according to the initial CAC score.

### 3.3. Comparison of Changes in CAC Scores and Risk Factors in the Initial Zero-CAC Group: Zero-CAC_last_ vs. New-CAC_last_

Of the 336 individuals with an initial CAC score of zero, 43 individuals (12.8%) developed coronary artery calcifications during follow-up (new-CAC_last_ group), whereas the remaining 293 individuals (87.2%) did not develop coronary artery calcifications (zero-CAC_last_ group).

Table 3 compares the changes in CAC scores and risk factors between these two groups. Age, body mass index (BMI), and hyperlipidemia prevalence were significantly higher in the new-CAC_last_ group than in the zero-CAC_last_ group (all *p* < 0.05). During follow-up, hypertension developed significantly more frequently in the new-CAC_last_ group; therefore, the prevalence rates of hypertension and hyperlipidemia at the time of the final CAC scan were higher in the new-CAC_last_ group than in the zero-CAC_last_ group (all *p* < 0.05).

### 3.4. Comparison of Changes in CAC Scores and Risk Factors in the Group with Initial CAC Presence: Rapidly vs. Slowly Progressing-CAC Groups

Among the 112 patients with CAC presence in the initial scan, 60 patients had rapid progression in CAC changes, with ΔCAC/year > 20; and 52 patients had slow progression, with ΔCAC/year ≤ 20. Table 4 compares the changes in CAC scores and risk factors between these two groups. BMI and prevalence of statin medication were higher in the rapidly progressing-CAC group than in the slowly progressing-CAC group (both *p* < 0.05). Likewise, the prevalence rates of newly developed hypertension and current smoking were higher in the rapidly progressing-CAC group than in the slowly progressing-CAC group (both *p* < 0.05). The initial CAC score was also higher in the rapidly progressing-CAC group than in the slowly progressing-CAC group (178.9 ± 190.5 vs. 38.5 ± 44.2, respectively; *p* < 0.001).

### 3.5. Predictors of Rapid CAC Progression

Table 5 shows the results of univariate and multivariate logistic regression analyses for the predictors of rapid CAC progression in asymptomatic adults. Based on univariate analysis, the parameters of age, male sex, BMI, and all risk factors including hypertension, DM, hyperlipidemia, current smoking, and family history of CVD were significantly associated with rapid CAC progression, defined as △CAC/year > 20 (all *p* < 0.05). Variables associated with a *p*-value < 0.05 in the univariate analysis were included in the multivariate regression. Using multivariate analysis, strong predictors for rapid CAC progression were the presence of hypertension at the time of the last CAC scan (odds ratio [OR] 11.3; 95% confidence interval [CI] 1.33, 95.42) and the initial CAC score (OR 1.05; 95% CI 1.04, 1.07).

## 4. Discussion

This study evaluated CAC progression according to changes in risk factors based on serial CTs to calculate the Agatston score in asymptomatic individuals. The main findings of the study were as follows: (1) At the time of the initial CAC scan, the parameters of age, prevalence of male sex, and all risk factors were significantly higher in the group with CAC presence than in the no-CAC group; (2) during the mean 3.5 ± 1.3 years of follow-up, 43 (12.8%) of 336 individuals with an initial CAC score of zero developed coronary artery calcifications, and 60 (53.6%) of the 112 individuals with initial CAC presence showed rapid annual CAC progression of more than 20; (3) new-onset hypertension is more frequently noted in the new-CAC_last_ group compared to in the zero-CAC_last_ group in the initial zero-CAC group, while newly developed hypertension and smoking were higher in the rapidly progressing-CAC group than in the slowly progressing-CAC group in the initial CAC presence group; (4) Age, male sex, BMI, and all risk factors were significantly associated with ΔCAC/year > 20, but recently diagnosed hypertension (OR, 11.3) and initial CAC score (OR, 1.05) were significant independent predictors in multivariate regression analyses. This suggests that patients with higher initial CAC scores may benefit from more frequent follow-up scans compared to patients with lower initial scores. This also suggests that continuously checking the changes in risk factors is important, because most risk factors are modifiable and dynamic in response to treatment.

Our study was motivated by the lack of guideline-supported recommendations for repeat CAC scans, although an increase in CAC score on serial scans is a well-known and strong predictor of CVD and worse survival rates [13]. Regarding the natural progression of CAC in asymptomatic populations with an initial CAC score of zero, limited prior studies have suggested not to repeat scans for at least 4–5 years because asymptomatic populations have a low incidence of CAC progression within 5 years [14,15,16]. Similarly, we observed a minimal increase (0.6 ± 2.7) in CAC score in the initial zero-CAC group during a mean follow-up of 3.5 years. However, considering that the rate of new-onset hypertension was higher in the new-CAC_last_ group than in the zero-CAC_last_ group, an interval of 4–5 years between the next follow-up CAC scans is sufficient when the initial CAC is zero, but it seems desirable to conduct a faster follow-up test when a new risk factor occurs.

Meanwhile, few studies have examined the natural progression of CAC in populations with an initial CAC score above zero, and no information exists on the optimal timing for follow-up scans in this population [17,18,19]. Based on several studies [13,20], it is evident that individuals with higher initial CAC scores tend to exhibit more rapid CAC progression over time. This finding is supported by the fact that persons with higher initial CAC scores have a higher risk of developing atherosclerosis and, hence, experience more pronounced CAC progression over time. Our study also demonstrated that the initial CAC score is one of the independent predictors for CAC progression, even after adjusting for traditional risk factors. Accordingly, the optimal timing and frequency of follow-up scans for this population should be decided based on their initial CAC score. Therefore, patients with pre-existing calcium in their coronary arteries should undergo follow-up CAC scans at intervals of no more than 3 years, to monitor CAC progression. However, due to the retrospective single-center study, it does not establish standardized guidelines for follow-up CAC.

Previous studies have demonstrated that traditional risk factors—such as age, male sex, BMI, hypertension, DM, hyperlipidemia, current smoking, and family history of CVD—are associated with the presence and degree of CAC progression [3], which is consistent with our results. Furthermore, Lehmann et al. [21] reported that recent risk factors assessed at the last follow-up—rather than initial risk factors—are the more influential predictors for coronary and cardiovascular event rates. Our study also demonstrates that new-onset hypertension and smoking was associated with rapid progression of CAC. This means that recent health conditions have the greatest impact on CAC progression because most clinical risk factors improve or worsen depending on lifestyle changes or medical treatment. Therefore, regular health checkups and risk factor control may be helpful for slowing down CAC progression.

Regarding medications, the effectiveness of lipid-lowering drugs—such as statins—in slowing the CAC progression rate has been a topic of considerable interest. While initial studies showed that CAC regressed with statin therapy, more recent data showed the opposite [22]. That is, recent studies have reported persistent CAC progression despite intensive lipid-lowering therapy [23,24]. Our study also identified statin medication as one of the independent predictors for CAC progression. This can be explained by the ‘calcium paradox’ concept of improved clinical outcome despite plaque progression when plaque progression is characterized by a predominant increase in dense calcium and plaque stabilization [25].

In terms of radiation exposure, the radiation dose of a CAC scan varied depending on the CAC scan protocol, the size of the individual’s heart, and CT equipment; but the average CAC scan has a radiation dose of 0.2–1 mSv, which is very low compared to contrast-enhanced coronary CT angiography (3–8 mSv). Therefore, CAC scans are recommended by the American Heart Association and the European Society of Cardiology as a screening tool for asymptomatic individuals. However, CAC scans are limited in assessing coronary artery stenosis and vulnerable plaques, which are composed of non-calcified lipid components. Therefore, early identification of rapid progression of CAC on serial CT scans offers an opportunity to highlight individuals with suspected coronary artery disease for a comprehensive CVD evaluation. This can facilitate early treatment for patients with coronary artery disease.

Our study has several limitations. First, our study has no information on cardiovascular events, as we aimed to assess CAC progression in relation to changes in risk factors. Second, we assessed major traditional risk factors such as hypertension and DM but did not include novel risk factors such as C-reactive protein or lipid profiles. Although there are no conclusive findings regarding their specific value for CAC progression, a prospective study would be needed to analyze more risk factors. Finally, as this is a single-center retrospective study using a single type of scanner, the generalizability of our findings to other populations and imaging settings may be limited. Hence, large-scale, multicenter, multivendor studies are needed to validate our results.

Future research should focus on validating whether repetitive CAC scanning can indeed reduce CVD incidence and mortality rates. Additionally, advancements in imaging techniques are necessary to decrease the cumulative radiation dose from repetitive CAC scanning.

In conclusion, our study showed that CAC progression was affected by demographic and traditional risk factors; but adjusting for these factors, recently diagnosed risk factors and the initial CAC score were the most influential factors for rapid CAC progression. This suggests that patients with higher initial CAC scores may benefit from more frequent follow-up scans and need continuous checks regarding changes in risk factors. Although our retrospective single-center study is limited in establishing clear guidelines, it is likely that individuals with zero CAC may not require follow-up for at least 4 years, while those with presence of CAC should be monitored at intervals shorter than 3 years. Furthermore, more frequent follow-up CAC scans may be needed when new risk factors develop. This personalized approach to follow-up could help optimize the timing of repeat scans and facilitate early detection and management of CVD.

## Figures and Tables

**Table 1:** Patient characteristics.

Parameter	Total (*n* = 448)	Zero-CAC (CAC = 0) (*n* = 336)	Low-CAC (0.1 ≤ CAC ≤ 100) (*n* = 70)	High-CAC (CAC > 100) (*n* = 42)
Initial CAC score	28.4 ± 93.2	0	31.6 ± 26.7 *	250.6 ± 190.3 *†
Age (years)	53.4 ± 8.1	51.9 ± 7.2	56.9 ± 9.4 *	57.4 ± 8.6 *
Male sex	329 (73.4%)	228 (67.9%)	62 (88.6%) *	39 (92.9%) *
BMI (kg/m^2^)	24.6 ± 2.9	24.4 ± 2.9	25.6 ± 2.6	24.7 ± 2.9
Risk factors				
Hypertension	90 (20.1%)	40 (11.9%)	21 (30.0%) *	22 (52.4%) *†
DM	29 (6.5%)	7 (2.1%)	11 (15.7%) *	11 (26.2%) *
Current smoker	159 (35.5%)	111 (33.0%)	30 (42.9%)	18 (42.9%)
Hyperlipidemia	77 (17.2%)	48 (14.3%)	19 (27.1%) *	12 (28.6%) *
Family history of CVD	111 (24.8%)	77 (22.9%)	24 (34.3%)	10 (23.8%)
Medication				
Statin	32 (7.1%)	12 (3.6%)	10 (14.3%) *	10 (23.8%) *
Aspirin	38 (8.5%)	14 (4.2%)	13 (18.6%) *	11 (26.2%) *
Number of risk factors				
None	154 (34.4%)	137 (40.8%)	11 (15.7%) *	6 (14.3%) *
1	174 (38.8%)	135 (42.0%)	23 (32.9%)	16 (38.1%)
≥2	120 (26.8%)	64 (19.0%)	35 (50.0%) *	21 (50.0%) *

BMI, body mass index; CAC, coronary artery calcium; CVD, cardiovascular disease; DM, diabetes mellitus. * *p*-value < 0.05 between the zero-CAC group vs. low-CAC group, or zero-CAC group vs. high-CAC group, from a pairwise comparison that used the zero-CAC group as a control. † *p*-value < 0.05 between low-CAC group vs. high-CAC group.

**Table 2:** Comparison of CAC progression and changes in risk factors according to the initial CAC score.

Parameter	Total (*n* = 448)	Zero-CAC (CAC = 0) (*n* = 336)	Low-CAC (0.1 ≤ CAC ≤ 100) (*n* = 70)	High-CAC (CAC > 100) (*n* = 42)
Follow-up CAC				
ΔCAC/year	10.0 ± 29.6	0.6 ± 2.7	19.7 ± 22.4 *	69.5 ± 64.3 *†
ΔCAC/year > 10	87 (19.4%)	6 (1.8%)	42 (60.0%) *	39 (92.9%) *†
ΔCAC/year > 20	61 (13.6%)	1 (0.3%)	26 (37.1%) *	34 (81.0%) *†
Follow-up risk factors				
Hypertension	103 (23.0%)	48 (14.3%)	29 (41.4%) *	25 (59.5%) *
New hypertension	14 (3.1%)	8 (2.4%)	8 (11.4%) *	3 (7.1%) *
DM	36 (8.0%)	8 (2.4%)	18 (25.7%) *	13 (31.0%) *
New DM	7 (1.6%)	1 (0.3%)	7 (10.0%) *	2 (4.8%)
Current smoker	137 (30.6%)	102 (30.4%)	23 (32.9%)	12 (28.6%)
New smoking	6 (1.3%)	0	4 (5.7%) *	2 (4.8%) *
Stop smoking	22 (4.9%)	9 (2.7%)	7 (10.0%) *	6 (14.3%) *
Hyperlipidemia	106 (23.7%)	65 (19.3%)	28 (40.0%) *	16 (38.1%) *
New hyperlipidemia	31 (6.9%)	17 (5.1%)	9 (12.9%) *	6 (14.3%) *

* *p*-value < 0.05 between the zero-CAC group vs. low-CAC group, or zero-CAC group vs. high-CAC group, from a pairwise comparison that used the zero-CAC group as a control. † *p*-value < 0.05 between low-CAC group vs. high-CAC group.

**Table 3:** Changes in CAC score and risk factors in the initial zero-CAC group.

Follow-Up CAC	Zero-CAC_last_ (n = 293)	New-CAC_last_ (n = 43)	*p*-Value
Age (years)	51.5 ± 6.9	54.6 ± 8.3	0.009 *
Male	194 (66.2%)	34 (79.1%)	0.115
BMI (kg/m^2^)	24.2 ± 2.9	25.3 ± 2.8	0.024 *
Initial risk factors			
Hypertension	31 (10.6%)	9 (20.9%)	0.073
DM	5 (1.7%)	2 (4.7%)	0.222
Current smoker	92 (31.4%)	19 (44.2%)	0.118
Hyperlipidemia	37 (12.6%)	11 (25.6%)	0.034 *
Family history of CVD	65 (22.2%)	12 (27.9%)	0.440
Medication statin	10 (3.4%)	2 (4.7%)	0.657
Medication aspirin	12 (4.1%)	2 (4.7%)	0.697
Follow-up risk factors			
Hypertension	35 (11.9%)	13 (30.2%)	0.004 *
New hypertension	4 (1.4%)	4 (9.3%)	0.011 *
DM	5 (1.7%)	3 (7.0%)	0.069
New DM	0	1 (2.3%)	0.128
Hyperlipidemia	49 (16.7%)	16 (37.2%)	0.003 *
New hyperlipidemia	12 (4.1%)	5 (11.6%)	0.052
Current smoker	86 (29.4%)	16 (37.2%)	0.292
New smoking	0	0	-
Stop smoking	6 (2.0%)	3 (7.0%)	0.095
Follow-up CAC score	0	16.7 ± 27.9	<0.001 *

* *p*-value < 0.05.

**Table 4:** Changes in CAC scores and risk factors in the group with initial CAC presence.

Follow-Up CAC Score	Slowly Progressing-CAC: ΔCAC/Year ≤ 20 (n = 52)	Rapidly Progressing-CAC: ΔCAC/Year > 20 (n = 60)	*p*-Value
Age (years)	59.3 ± 9.5	56.4 ± 8.5	0.091
Male sex	46 (88.5%)	55 (91.7%)	0.752
BMI (kg/m^2^)	24.5 ± 2.6	25.9 ± 2.8	0.010 *
Initial risk factors			
Hypertension	15 (28.8%)	28 (46.7%)	0.079
DM	9 (17.3%)	13 (21.7%)	0.638
Current smoker	19 (36.5%)	29 (48.3%)	0.252
Hyperlipidemia	12 (23.1%)	22 (36.7%)	0.398
Family history of CVD	65 (22.2%)	12 (27.9%)	0.150
Medication statin	5 (9.6%)	15 (25.0%)	0.047 *
Medication aspirin	10 (19.2%)	14 (23.3%)	0.650
Follow-up risk factors			
Hypertension	35 (30.8%)	38 (63.3%)	0.001 *
New hypertension	1 (1.9%)	10 (16.7%)	0.010 *
DM	11 (21.2%)	20 (33.3%)	0.204
New DM	2 (3.8%)	7 (11.7%)	0.172
Hyperlipidemia	18 (34.6%)	26 (43.3%)	0.438
New hyperlipidemia	6 (11.5%)	9 (15.0%)	0.782
Current smoker	13 (25.0%)	22 (36.7%)	0.222
New smoking	0	6 (10.0%)	0.029 *
Stop smoking	6 (11.5%)	7 (11.7%)	>0.99
CAC score			
Initial CAC score	38.5 ± 44.2	178.9 ± 190.5	<0.001 *
Follow-up CAC score	66.7 ± 57.3	434.8 ± 408.6	<0.001 *
ΔCAC/year	7.7 ± 7.0	64.9 ± 54.6	<0.001 *

* *p*-value < 0.05.

**Table 5:** Predictors for an annual CAC progression of more than 20.

Parameter	Rapid CAC Progression (ΔCAC/year > 20)			
	Univariate OR (95% CI)	*p*-Value	Multivariate OR (95% CI)	*p*-Value
Age	1.05 (1.02, 1.08)	0.003 *	0.96 (0.89, 1.03)	0.206
Male sex	4.67 (1.83, 11.98)	0.001 *	1.15 (0.19, 6.86)	0.876
BMI	1.18 (1.08, 1.30)	<0.001 *	1.30 (1.04, 1.63)	0.051
Initial risk factors				
Hypertension	5.12 (2.87, 9.14)	<0.001 *	0.22 (0.02, 1.97)	0.174
DM	6.28 (2.85, 13.85)	<0.001 *	0.20 (0.02, 1.87)	0.157
Hyperlipidemia	2.71 (1.49, 4.95)	0.001 *	1.77 (0.28, 11.20)	0.545
Current smoking	1.94 (1.12, 3.34)	0.017 *	0.66 (0.14, 3.18)	0.606
Family history of CVD	1.89 (1.06, 3.35)	0.03 *	1.74 (0.55, 5.46)	0.344
Medication				
Statin	7.09 (3.32, 15.16)	<0.001 *	1.61 (0.34, 7.50)	0.546
Aspirin	4.50 (2.18, 9.31)	<0.001 *	0.56 (0.16, 2.01)	0.376
Follow-up risk factors				
Hypertension	8.34 (4.65, 14.94)	<0.001 *	11.30 (1.33, 95.42)	0.026 *
New hypertension	8.24 (3.20, 21.23)	<0.001 *		
DM	9.45 (4.66, 19.14)	<0.001 *	5.95 (0.79, 44.93)	0.084
New DM	16.59 (4.16, 66.10)	<0.001 *	-	
Hyperlipidemia	2.95 (1.69, 5.18)	<0.001 *	0.99 (0.19, 5.13)	0.995
New hyperlipidemia	2.74 (1.20, 6.24)	0.016 *		
Smoking	1.45 (0.83, 2.54)	0.196	4.18 (0.78, 22.54)	0.096
New smoking	0	-		
Initial CAC score	1.04 (1.03, 1.05)	<0.001 *	1.05 (1.04, 1.07)	<0.001 *
0	1	(ref)	1	(ref)
0.1–100	0.001 (0, 0.01)	<0.001 *	0 (0, 0.01)	<0.001 *
>100	0.14 (0.06, 0.35)	<0.001 *	0.06 (0.02, 0.19)	<0.001 *

BMI, body mass index; CAC, coronary artery calcium; CI, confidence interval; CVD, cardiovascular disease; DM, diabetes mellitus; OR, odds ratio. * *p*-value < 0.05.

## Data Availability

The data that support the findings of this study are available from the corresponding author, Eun Ju Chun, upon reasonable request.
